# Design, Analysis, and Manufacturing of Diffractive Achromatic Optical Systems

**DOI:** 10.3390/mi16030322

**Published:** 2025-03-11

**Authors:** Yidi Zheng, Junfeng Du, Boping Lei, Jiang Bian, Lihua Wang, Bin Fan

**Affiliations:** 1 National Key Laboratory of Optical Field Manipulation Science and Technology, Chinese Academy of Sciences, Chengdu 610209, China; 2 Institute of Optics and Electronics, Chinese Academy of Sciences, Chengdu 610200, China; 3 University of Chinese Academy of Sciences, Beijing 100049, China

**Keywords:** diffractive optical elements, chromatic aberration, Schupmann achromatic model

## Abstract

The increasing resolution requirements of imaging optical systems must be satisfied by expanding the aperture of the optical system according to Rayleigh’s criterion, and larger apertures of conventional refractive/reflective optics place a greater demand on manufacturing and transportation. Diffractive optics are applied to imaging optics to achieve lightweight design, but the image quality suffers due to their strong negative properties. Therefore, a wide-band imaging system based on the Schupmann achromatic model is proposed in this paper to solve the above problem, and the achromatic performance of the system is guaranteed by the Schupmann achromatic model. The aperture of the relay lens is reduced, since using harmonic diffractive optics as the primary lens results in a much more compact focus compared to the diffractive optics in the same wavelength band. This allows for the lightweight design of the optical system. An 80 mm aperture diffractive optical system covering the 400–900 nm band was designed and fabricated to verify the above theory. The actual resolution of the optical system was 76.196 lp/mm, and the achromatic task was accomplished. The design and experimentation of the wide-band achromatic imaging optical system confirms that the proposed theory is correct, and lays the foundation for the further application of large aperture diffractive telescopes.

## 1. Introduction

The resolving power of imaging optical systems, such as telescopes, increases with aperture size, according to Rayleigh’s criterion [1,2,3]. However, strict surface tolerance, high-precision machining and mounting, and difficult transportation and deployment limit the further increase of the aperture of reflective imaging optical systems. As researchers continue to explore new imaging optical systems, diffractive imaging optical systems have increasingly entered their field of vision. These systems use diffractive optical elements as the primary lenses, effectively solving the above problems for researchers.

Diffractive optical elements (DOE) are maturing and are being employed in a wide range of applications due to the rapid advancement of microfabrication technology [4,5,6,7,8]. This form of optical element has a stronger phase modulation function than conventional refractive/reflective optics. This function is achieved by using micro-nano structures with varying heights on their surfaces. The diffractive primary lens will be lightweight and easy to unfold because the micro-nano structures can be fabricated on planar thin films or thin substrate materials, whereas the diffractive primary lens as a transmitting optic element allows for more relaxed large aperture splicing tolerances than the reflective primary lens. The advantages listed above provide the possibility for further large aperture space telescope design and fabrication.

DOEs, on the other hand, have a theoretical 100% diffraction efficiency only at the design wavelength, and they have a strong negative chromatic dispersion property, i.e., the direction of chromatic dispersion is the opposite of that of the refractive lens, which severely limits their use in wide-band imaging optical systems. Researchers have created harmonic diffractive optical elements (HDOE) [9,10,11] as well as multilayer diffractive optical elements (e.g., dual and multi-layers) [12,13,14] to address the issue of a decrease in diffraction efficiency when the operating wavelength differs from the design wavelength. The greater microstructure height of HDOE results in additional harmonic wavelengths with high diffraction efficiency; nonetheless, their potential to improve diffraction efficiency in the wide band remains limited. Dual (multi-) layer diffractive optics use a combination of diffractive optics from different materials to achieve the task of high diffraction efficiency in the wide band, while retaining the flat image field property of diffractive optics. However, the design of dual (multi-)layer diffractive optics places high demands on material selection and optimization methods, and processing problems such as element eccentricity still need be solved. Computational imaging techniques incorporating (H)DOE have recently been emerging as novel solutions for high-quality diffractive imaging, but their application in very large aperture telescopes remains to be developed.

The Schupmann achromatic system was initially used in refractive optics, but was later extended to diffractive optics. The system primarily employs a diffractive primary lens and a diffractive secondary lens located at its conjugate position, which possesses the same dispersion as the diffractive primary lens, but opposite focal power, to accomplish the achromatic task. The Schupmann achromatic system is a mature achromatic diffractive imaging optical system, and there are already examples of large aperture diffractive achromatic applications with significant reference value.

Hyde et al. of the Lawrence Livermore National Laboratory (LLNL) presented research on very large (25–100 m) telescopes employing DOE [15,16], and a wide-band imaging method for large aperture telescopes based on the Schupmann achromatic model was also proposed. The US Defense Advanced Research Projects Agency (DARPA) carried out the “MOIRE” diffractive thin-film imaging project [17,18,19], in which a diffractive space telescope with an aperture of 10 m to 20 m and a bandwidth of 70 nm will be created in stages throughout the program. In 2010, Koechlin et al. developed a diffractive imaging optical system with a 200-mm aperture Fresnel lens as the primary lens [20], and the group realized imaging observations of targets such as Saturn in the spectral range of 630–743 nm with a spectral width of 113 nm. Andersen et al. designed a telescope imaging optical system with an aperture of 200 mm in the spectral range of 522–562 nm [21] and a bandwidth of 40 nm, with a photon sieve as the primary lens.

However, the main lens of the above imaging optical systems are all conventional Fresnel diffractive structure lenses or photon sieves, and although the use of these two devices makes the optical system more lightweight, the strong chromatic aberration makes it difficult to realize wide-band imaging with the large-diameter diffractive main lens, and the application of diffractive imaging telescopes is limited due to their narrow spectral range, which is the main problem limiting the further development of diffractive telescopes at present. It is therefore necessary to study how to make wide-spectrum large aperture diffraction achromatic imaging more lightweight.

To address the aforementioned issues, a broadband diffractive imaging optical system based on an achromatic structure is proposed in this paper. The optical system, incorporating the Schupmann achromatic structure, has been optimized and designed. The primary distinction between the imaging optical system proposed in this paper and the aforementioned imaging optical systems lies in the use of a HDOE as the primary lens. The main focus of this work is to achieve achromatic performance and lightweight design of the imaging optical system while operating over a broad bandwidth. Firstly, the Schupmann achromatic model ensures the achromatic performance of the system, and the system bandwidth is increased due to the use of HDOE as the primary lens. The spectral bandwidth of the optical system covers the 400–900 nm range. Secondly, the smaller dispersion range of HDOE enables a reduction in the aperture of the system’s relay lens compared to using a DOE, thus achieving a lightweight system compared to using a DOE as the primary lens. Ultimately, the organic combination of a new optical imaging device and an achromatic theoretical model is realized in this paper.

## 2. Principles and Design Methods

### 2.1. Principle of Schupmann Achromatic Model

**Figure 1:**
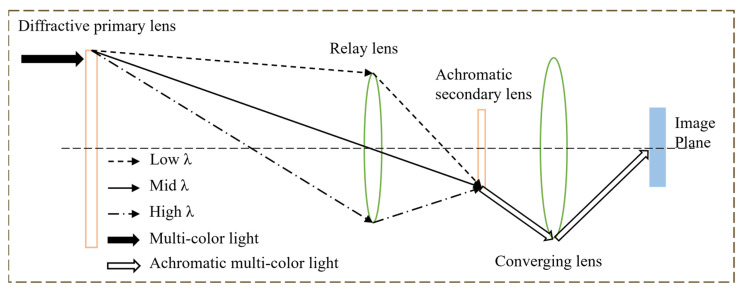
Schupmann achromatic model.

As mentioned in Section 1, DOE has a strong negative dispersion, necessitating the optical design of achromatic imaging to provide diffractive broadband imaging. The Schupmann achromatic optical system is the most effective achromatic structure for a large-diameter diffractive primary lens imaging optical system. As shown in Figure 1, the optical system consists of a diffractive primary lens, a relay lens, a diffractive secondary lens, and a converging lens. Among them, the diffractive primary lens and diffractive secondary lens are in the conjugate position of the relay lens and have the same dispersion and opposite focal power, so that the wide band light can complete the achromatic function after the primary lens, relay lens and achromatic secondary lens. The convergence lens also plays a very critical role, since the focal power of the optical system is negative after achromatic imaging is completed, necessitating the use of a convergence lens to bring the light together and eventually achieve achromatic imaging.

For the diffracting primary lens and achromatic diffracting lens, the focal power of both should satisfy the following relationship:

(1) $$\frac{1}{-\alpha^{2}}=\frac{\varphi_{N}}{\varphi_{1}},$$

where *α* is the magnification of the relay system. Then, the relationship between the diffractive primary lens and the diffractive achromatic secondary lens is as follows:

(2) $$-\alpha^{2}=\frac{f_{N}}{f_{1}},$$

(3) $$\alpha =\frac{{F_{N}}^{\#}}{{F_{1}}^{\#}}=\frac{l_{N}}{l_{1}},$$

where $f_{N}$ and $f_{1}$ are the focal lengths of the diffractive primary lens and the diffractive achromatic secondary lens, respectively. ${F_{N}}^{\#}$ and ${F_{1}}^{\#}$, $l_{N}$ and $l_{1}$ represent the F-number of the diffracting primary lens and diffracting achromatic secondary lens, as well as the distances from the diffracting primary lens to the relay lens and the relay lens to the diffracting achromatic secondary lens, respectively.

The Schupmann achromatic model can achieve 400–900 nm or an even wider band imaging in theory, but due to the presence of chromatic aberration in the primary lens, as shown in Figure 1, the aperture of the relay lens needs to be increased along with the increase in the spectral range of the system. The aperture of the relay lens will be greater than 4 m according to the calculations of the primary lens aperture of 10 m and the spectral coverage of the 400–900 nm. This leads to issues such as increased weight and mounting accuracy of the optical system, which is incompatible with the optical system’s original design and construction purpose. Therefore, adjusting the aperture of the relay lens can significantly lessen the difficulty of realizing the diffractive large-aperture telescope using the Schupmann achromatic model.

### 2.2. Principles of DOE and HDOE

**Figure 2:**
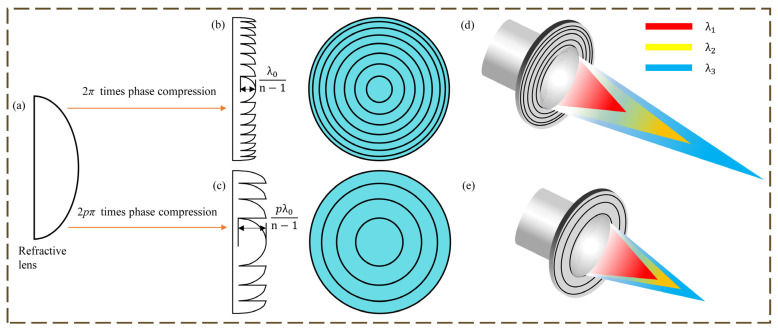
(**a**) Ideal refractive lens; (**b**) Schematic diagram of DOE; (**c**) Schematic diagram of HDOE; (**d**) Schematic diagram of the dispersion of the DOE; (**e**) Schematic diagram of the dispersion of the HDOE.

The difference between DOE and HDOE is shown in Figure 2. The greater depth of the microstructure and the smaller number of ring bands are the surface manifestations of HDOE compared to DOE. HDOE is a broad expression of DOE. In fact, the phase-depth factor, which is the difference between the two, is the key parameter in diffractive optics design. The phase-depth factor of HDOE is *p* (*p* is an integer greater than 1 and is also known as the diffraction order), and the diffractive plane is the high-order diffractive plane (HODS), whereas the conventional DOE is 1 and has a first-order diffractive surface (FODS). While the theoretical 100% diffraction efficiency can only be attained at the DOE’s design wavelength, the HDOE can be obtained at both the design wavelength and at multiple harmonic wavelengths, significantly improving the imaging performance of the HDOE in broadband imaging optics [9].

A conventional refractive lens goes through a phase compression operation to obtain the DOE or HDOE, which is given by:

(4) $$\varphi_{doe}=mod\left(\varphi_{lens},p\times 2\pi \right),$$

where $\varphi_{doe}$ represents the compressed phase, $\varphi_{lens}$ represents the continuous phase of the refractive lens, *mod* is a remainder operation with $\varphi_{lens}$ as the dividend, and *p* × 2π is the divisor.

The height of the microstructures can be obtained from the phase versus optical range:

(5) $$\varphi_{lens}=\left(n-1\right)\times d\times \frac{2\pi }{\lambda },$$

where *n* is the refractive index of the lens material at the operating wavelength and *d* is the height of the microstructure of the diffractive optical element.

For a system that images objects in the visible spectrum (400–700 nm) with a center wavelength of 550 nm, a DOE with *M* = 1 exhibits > 50% Δf change in focus. The resulting blur creates unacceptable images without correction. High harmonic lens systems have considerably less focal dispersion. For example, an *M* = 250 system used for the visible spectrum exhibits only a 0.4% change in focus and is apochromatic in the sense that there are multiple wavelengths *λ_p_* that focus to *f*_0_ given by:

(6) $$\lambda_{p}=M\lambda_{0}/p,$$

where *M* is the focal diffraction order at the design wavelength, and the Δf change is limited to approximately *f*_0_/*M*.

The diffractive lens is used as the primary lens to design a set of imaging optical systems with a spectral range of (*λ_min_*, *λ_max_*), where *λ*_1_, *λ*_2_, *λ*_3_ are the three wavelengths in the spectral range as shown in Figure 2, the relationship of *λ*_1_, *λ*_2_, *λ*_3_ satisfies Equation (7), and *q*, *m* and *n* are integers larger than 1.

(7) $$q\lambda_{1}=m\lambda_{2}=n\lambda_{3}.$$

When the primary lens is DOE, the phase of each ring-band cycle is 2π. Utilizing first-order diffraction imaging, the focal points of wavelengths *λ*_1_, *λ*_2_, and *λ*_3_ are shown in Figure 2. The relationships between the different focal lengths *f*_1_, *f*_2_, and *f*_3_ corresponding to the wavelength *λ*_1_, *λ*_2_, and *λ*_3_ can be expressed by Equation (8). Both Equation (8) and Figure 2 shows a significant chromatic aberration of DOE.

(8) $$\lambda_{1}f_{1}=\lambda_{2}f_{2}=\lambda_{3}f_{3}.$$

When the primary lens is HDOE, as mentioned above, each of its ring-band cycles is in phase 2 *p*π (*p* is an integer with *p* ≥ 1), the design spectral wavelengths *λ*_1_, *λ*_2_, and *λ*_3_ satisfy Equation (7), and the focal lengths of the *q*, *m*, and *n* diffraction order of the HDOE at *λ*_1_, *λ*_2_, and *λ*_3_ overlap and have equal focal lengths with the relationship, as shown in Equation (9).

(9) $$f_{1}=f_{2}=f_{3}.$$

With *λ*_1_, *λ*_2_, and *λ*_3_ as the center wavelength, the spectral range is (*λ*_1*min*_, *λ*_1*max*_), (*λ*_2*min*_, *λ*_2*max*_), (*λ*_3*min*_, *λ*_3*max*_) spectral segments (in which *λ*_1*min*_ = *λ_min_*, *λ*_3*max*_ = *λ_max_*). The three spectral segments have the same optical path and basically overlap the focal point. At this time the color difference of HDOE is equivalent to about 1/3 of DOE, and it is only necessary to use the Schupmann achromatic model to finish the achromatic design of one of the spectral segments. The two remaining spectral segments will achieve achromatic imaging at the same time due to the use of the same optical path for the three spectral segments. Through the reasonable design of the center wavelength, when *λ*_1*max*_ = *λ*_2*min*_ and *λ*_2*max*_ = *λ*_3*min*_, the continuous broadband imaging in the spectral range (*λ_min_*, *λ_max_*) is realized. The aperture of the relay lens will be greatly reduced due to the fact that the HDOE chromatic aberration is only about 1/3 of the DOE chromatic aberration. This is accompanied by a great degree of reduction in the difficulty in developing the system, due to a significant reduction in the weight of the system.

The above example selects three spectral bands in the spectral range (*λ_min_*, *λ_max_*). The chromatic aberration of HDOE will be further reduced and the aperture of the relay lens will continue to decrease when the number of spectral bands increases, but the increase of spectral bands also brings new problems. The analysis and selection of the number of spectral bands will be carried out in Section 2.3.

### 2.3. Methodology for the Design of Broad-Band Diffractive Imaging Optical Systems

The design method of the diffraction imaging optical system with HDOE as the primary lens will be presented in this section, which is based on the theory and analysis above.

Firstly, the center wavelength of the optical system is selected. The center wavelength is usually chosen to be 632.8 nm for visible imaging optical systems, which helps the interferometer accomplish the detection task because the interferometer wavelength is usually 632.8 nm. According to the calculation of HDOE, the different harmonic wavelengths are chosen to be the center wavelengths of the corresponding wavelength bands, resulting in overlapping focal points when imaged through the diffractive achromatic imaging optical system.

Secondly, about the choice of the harmonic diffraction order, theoretically, the larger the diffraction order *p* is, the more harmonic wavelengths there are in the imaging range, and the narrower the spectral range covered by each band, so the aperture of the relay lens will be smaller. However, as the diffraction order *p* increases, so does the thickness of the HDOE microstructure. The stronger refractive effect of the lens introduces the remainder of the diffraction order into the aberration, reducing the optical system’s imaging performance. Therefore, the maximum range of the diffraction order *p* should be calculated according to the microstructure thickness on the remaining diffraction order wavelength. The introduced optical range difference PV value is less than 1/4 times of the wavelength.

With $\lambda_{p}$ as the center wavelength, the highest depth of the HDOE microstructure is given by:

(10) $$h=\frac{p\lambda_{p}}{n(\lambda_{p})-1},$$

where $n(\lambda_{p})$ is the refractive index of the substrate material at the wavelength $\lambda_{p}$. Then, the wavefront optical range difference at the corresponding harmonic length $\lambda_{m}$ is given by:

(11) $$w=\frac{p\lambda_{p}[n(\lambda_{m})-1]}{n(\lambda_{p})-1}-m\lambda_{m}.$$

Since wavelength $\lambda_{m}$ is the harmonic length of wavelength $\lambda_{p}$, there is:

(12) $$w=\frac{m\lambda_{m}[n(\lambda_{m})-n(\lambda_{p})]}{n(\lambda_{p})-1}.$$

According to the Rayleigh’s criterion, the wavefront optical range difference of an optical system with excellent imaging quality should be satisfied:

(13) $$w\leq \frac{\lambda_{m}}{4}.$$

If refractive indexes of certain characteristic line spectrums are given, the empirical formula of the refractive index with the change of wavelength for this optical material can be expressed as [22]:

(14) $$n\left(\lambda \right)=n_{A}B_{A}+n_{C}B_{C}+n_{F}B_{F}+n_{h}B_{h},$$

where, $n_{A}$, $n_{C}$, $n_{F}$ and $n_{h}$ are the refractive indexes of the characteristic line spectrums $\lambda_{A}$ = 0.7682 μm, $\lambda_{C}$ = 0.65628 μm, $\lambda_{F}$ = 0.48613 μm, and $\lambda_{h}$ = 0.40466 μm, respectively.

The calculations for $B_{A}$, $B_{C}$, $B_{F}$ and $B_{h}$ can be found in reference [22]. According to the aforementioned formula, the chromatic dispersion of the material may cause wavefront aberration in the HDOE design process. Combining Equations (13) and (14), the HDOE design should satisfy the following equation:

(15) $$\left|\frac{d\left(n-1\right)}{p\lambda }-1\right|\leq \frac{1}{4},$$

In the spectral range of 400–900 nm for the band of interest in this paper, the center wavelength $\lambda_{p}$ is 632.8 nm when $\lambda_{m}$ is 400 nm. When the substrate material is quartz material, $n(\lambda_{m})$ is 1.47011, and $n(\lambda_{p})$ is 1.45702. Substituting into the above equation, the diffraction order *p* is maximized to be 5. Based on the above conclusion, the enhancement of the refractive properties of HDOE will degrade the image quality of the system when the diffraction order *p* is further increased. Therefore, it should be ensured that the diffraction order *p* ≤ 5 is 632.8 nm.

Comparing with traditional diffraction achromatic imaging, theoretically, the broad band imaging of 400–900 nm can be realized by adopting DOE instead of HDOE as the primary lens. The achromatic optical imaging systems of HDOE and DOE as the primary lens are set to have an aperture of 10 m and an F-number of 10 according to the calculation. The aperture of the relay lens at 100 m from the primary lens is about 1.9 m and 4.3 m, respectively, and at this time, the ratio of the aperture of the relay lens, when DOE and HDOE are used as the primary lenses, respectively, is about two times. In other words, the use of HDOE as the primary lens can ensure the wide-band imaging of the system while reducing the aperture of the relay lens. This can greatly reduce the weight of the imaging optical system, especially when expanding to the application of a large aperture optical system, which is very important because the system provides a higher engineering feasibility.

## 3. Optical Design and Experimentation

### 3.1. Optical Design

In order to verify the design method and imaging effect of the harmonic diffraction broad spectrum imaging optical system, a set of imaging optical systems with an aperture of 80 mm and a spectral range of 400–900 nm is designed in this section. The design method of this optical system is consistent with the design method introduced in Section 2.

The center wavelength is selected as 632.8 nm, and the diffraction order at the center wavelength of 632.8 nm is designed as three-order. The rest of the diffraction orders are 949.2 nm diffraction order 2, 476.4 nm diffraction order 4, and 379.7 nm diffraction order 5, and the corresponding spectral bandwidths are 400–420 nm, 420–550 nm, 550–750 nm, and 750–900 nm, respectively.

The F-number of the diffracting primary lens is 10, and the ratio of the F-number of the diffracting primary lens to that of the achromatic diffracting secondary lens was set to 1.33. Adopting the Schupmann achromatic model, the system consists of a primary lens, a relay mirror, a reversing mirror, a converging mirror, and a harmonious diffractive achromatic secondary lens. The optimization process requires attention to the following: first, keep the phase coefficients of the primary lens unchanged; second, control the size of the optical elements, such as the relay structure and the diffraction achromatic secondary lens, in order to reduce the weight and the machining pressure; third, control the apertures of the optical elements, the pitch, etc., to achieve smooth transmission of light and to reduce the tolerance sensitivity; fourth, use the lens to control the angle of light dispersion in the immediate rear of the diffraction achromatic secondary lens, because the diffraction achromatic secondary lens has a strong light deflection ability, which tends to disperse light, and the light passes through the diffraction achromatic secondary lens twice through the converging mirror. This allows for an increase in the size of the feature to reduce processing difficulties.

**Figure 3:**
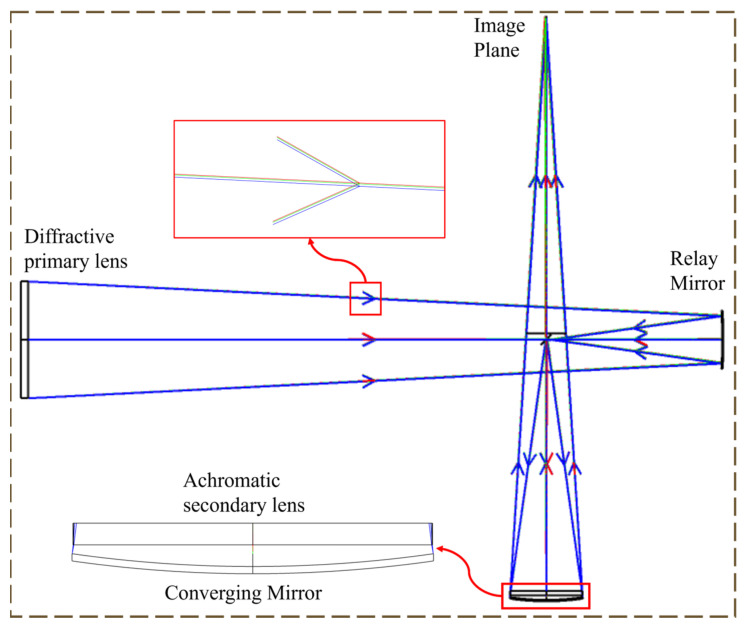
Schematic diagram of the optical system, where the green arrow, the red arrow, and the blue arrow represent the direction of propagation of the light rays at 0°, 0.112°, and 0.16° of the field of view, respectively.

The final optimized system optical path is shown in Figure 3. The system parameters are shown in Table 1. The system has an aperture of 80 mm, an imaging spectral range of 400–900 nm, a full field of view of 0.16°, and a focal length of 656.2 mm. The off-axis design is used in this paper for two main purposes. One is that the optical system is more compact due to the saving of axial space, and the second is that the only lenses in the system are the primary lens and the diffractive achromatic secondary lens. The mirrors do not introduce additional chromatic aberrations to the optical system, which achieves the proposed achromatic aberration objective of this paper.

In the design of the 80 mm experimental system, the diffraction order of the primary lens is set to 3, because the number of steps of HDOE increases with the increase of the diffraction order, under the premise of guaranteeing the diffraction efficiency. Considering that the diffraction efficiency is more than 80%, when the diffraction order is set to 3, the number of steps only needs to be more than 12. When the diffraction order is set to 5, the number of steps needs to be more than 20, which raises the difficulty of the actual processing of HDOE. Considering the existing processing order, it is reasonable and appropriate to choose three diffraction steps at 632.8 nm for the 80 mm aperture experiment. Table 2 shows the data for the harmonic diffraction primary lens.

According to the formula for the microstructure height of HDOE shown in Figure 2c, the microstructure height of HDOE with 632.8 nm as the center wavelength and diffraction order *p* = 3 is about 4.1539 μm in this paper. After bringing the data in Table 3 into Equation (15), it is found that the wavefront error of HDOE has very little effect on the actual imaging even though the chromatic dispersion of the material causes additional foci and displacement of the main focus.

**Figure 4:**
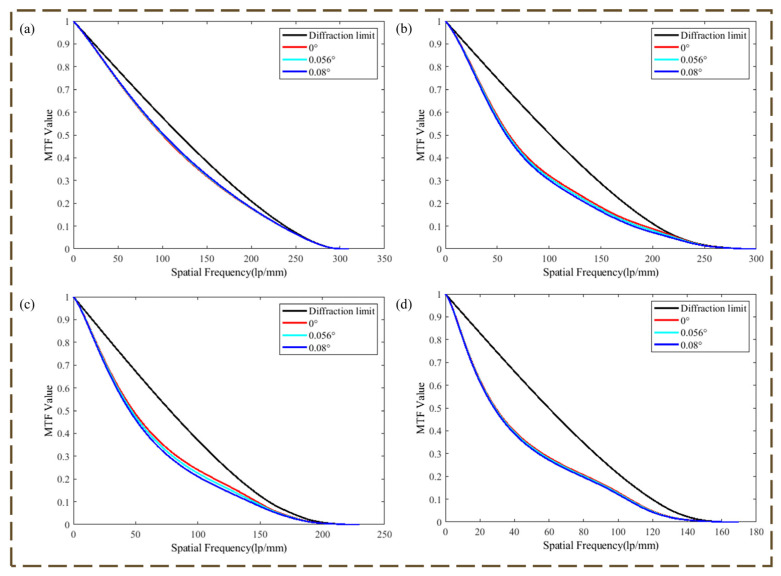
MTF at each spectral band of the optical system. (**a**) MTF of optical system at 400–420 nm; (**b**) MTF of optical system at 420–550 nm; (**c**) MTF of optical system at 550–750 nm; (**d**) MTF of optical system at 750–900 nm.

The MTF performance of the optical system is shown in Figure 4.

An 80-mm aperture spectral range 400–900 nm wide-band imaging system was designed using a Schupmann achromatic model, with MTF mid-range frequencies decreasing due to center blocking.

### 3.2. Experiment of Optical Imaging

**Figure 5:**
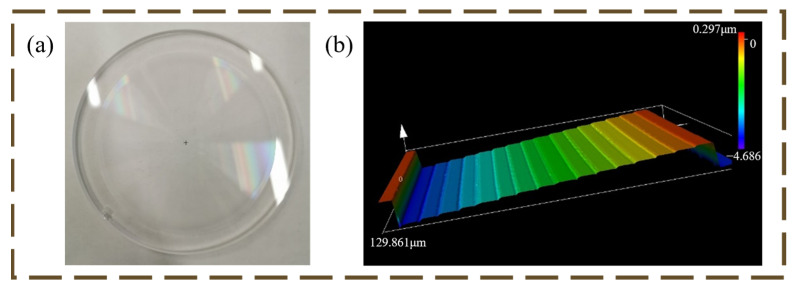
(**a**) Physical diagram of HDOE; (**b**) Harmonic diffractive lens structure machined as 16-step.

**Figure 6:**
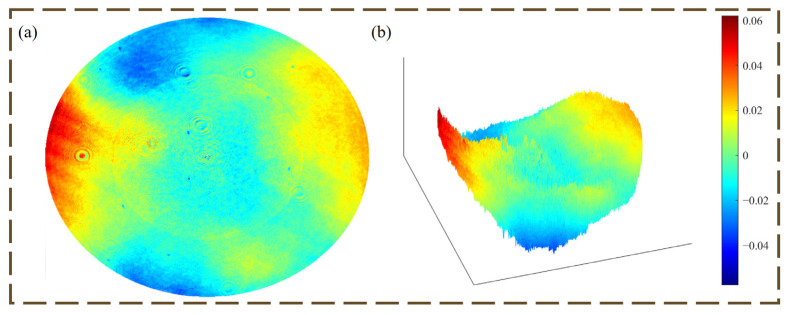
Transmitted wavefront of the primary lens of a diffractive imaging optical system (**a**) Top view of the transmitted wavefront; (**b**) Three-dimensional view of the transmitted wavefront.

The microstructure of the primary lens was fabricated on a quartz substrate, and the 16-step microstructure ensures the high diffraction efficiency of the primary lens, which is physically shown in Figure 5a. Figure 6 shows the transmitted wavefront detected by an interferometer after the completion of the primary lens processing. The wavefront RMS is about 0.049λ (λ = 632.8 nm).

**Figure 7:**
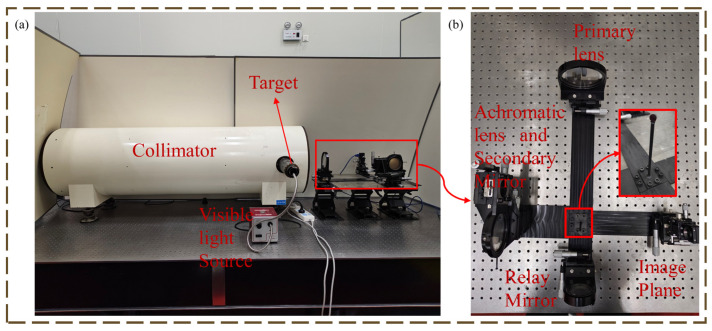
(**a**) Schematic diagram of the experiment; (**b**) Physical diagram of the optical system.

**Figure 8:**
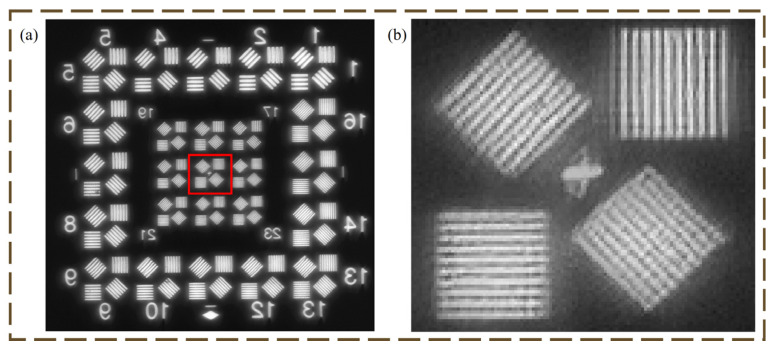
Resolution target imaging; (**a**) Imaging results of the achromatic optical system proposed in this paper; (**b**) Imaging results of group 25.

The experimental imaging optical path is shown in Figure 7. A halogen lamp is used as a visible light source. Its spectral range covers 200 nm to 2500 nm to meet the requirements of the optical system in this paper. The target is placed at the focal plane of the parallel collimator to provide an infinity target. The imaging is as shown in Figure 8.

The 25th group of the resolution target A2 can be resolved as shown by Figure 8b, which greatly verifies the achromatic capability of the imaging optical system proposed in this paper. The 25th group of the A2 resolution target corresponds to a line width of *d* = 20 μm, and the resolution of the optical system can then be computed according to the following equation:

(16) $$\frac{d}{f_{\mathrm{collimator}}}=\frac{x}{f_{\mathrm{system}}},$$

where *x* is the minimum distance that the optical system can resolve at the image plane. The relationship with the resolution of the optical system $R_{o}$ is:

(17) $$R_{o}=\frac{1}{2x},$$

where the focal length of the parallel collimator $f_{\mathrm{collimator}}$ = 2 m, $f_{\mathrm{system}}$ for the focal length of the optical system is 656.2 mm as shown in Table 1, and *x* for the object surface to resolve the minimum distance *d* corresponds to the image surface of the image surface resolvable distance. The substitution of Equation (14) can be understood as *x* = 0.0064 mm, so the actual resolution of the optical system $R_{o}$ = 76.196 lp/mm, which is completely acceptable for a visible light optical system. Although the cost of obtaining such high quality images is that the system is used in a narrow angular field of view, this does not affect the design and performance verification of the lightweight achromatic diffraction imaging system in this paper.

The achromatic model as well as imaging performance reliability and validity of the proposed optical system design method is demonstrated in this section.

## 4. Conclusions

In conclusion, an optical system based on the HDOE as a primary lens combined with the Schupmann achromatic model is proposed to accomplish the achromatic design of a broad-spectrum diffractive imaging optical system, and to make the system more lightweight. The optical system uses the HDOE, which has the same diffraction order as the primary lens, as an achromatic secondary lens to accomplish the achromatic task, and the HDOE as the primary lens reduces the aperture of the relay mirror while accomplishing a part of the lightweighting task, thus making the optical system more lightweight. The aperture of the relay mirror has a 50% reduction for a theoretical 10 m aperture optical system based on simulations. For an experimentally validated 80 mm aperture optical system in this paper, an off-axis design is used in order to save axial space. The image achromatic performance of the optical system is very significant and actually has a 76.196 lp/mm, even though the theoretical MTF mid-range frequencies of the system decrease due to the case of center blocking. The above results demonstrate that the proposed method of using HDOE as a wide-band achromatic diffraction imaging optical system is feasible and effective. The system still has a strong potential to combine with image processing to achieve further resolution enhancement. This is due to less pressure on the back-end image processing because of the better achromatic performance of the optical imaging system, which provides a way to design and realize future wide-band large aperture diffractive space telescopes.

## Figures and Tables

**Table 1:** Data for the optical system.

Wavelength	Field of View	Aperture	Focal Length	F/#
400–900 nm	0.16°	80 mm	656.2 mm	8.18

**Table 2:** Data for the diffractive primary lens.

Thickness	Aperture	A2	A4	A6	A8	A10
5 mm	80 mm	−2.071	8.11 × 10^−7^	−6.35 × 10^−13^	6.56 × 10^−19^	−9.36 × 10^−24^

A2 is the second-term coefficient of HODS. A4 is the fourth-term coefficient of HODS. A6 is the sixth-term coefficient of HODS. A8 is the eighth-term coefficient of HODS. A10 is the tenth-term coefficient of HODS.

**Table 3:** Spectral data for the optical system.

Number	Center Wavelength	Diffraction Order	Spectral Range	Bandwidth
1	946.4 nm	2	750–900 nm	150 nm
2	632.8 nm	3	550–750 nm	200 nm
3	476.4 nm	4	420–550 nm	130 nm
4	379.7 nm	5	400–420 nm	20 nm

## Data Availability

The data are available within the article.
